# Medical Items

**Published:** 1894-06

**Authors:** 


### *MEDICAL ITEMS.*

---

BEFORE the late meeting of the Virginia Examining Board there were seventy-two applicants for license. Twenty of these were rejected, or 27.5 per cent.

---

THE following medical journals have ceased publication, viz.: *The National Medical News, The Epitome of Medicine, The Country Doctor, The Weekly Medical Bulletin* of Chicago, *The American Gynecological Journal* of Toledo, and the *Journal of Surgery, Gynecology and Obstetrics* of Atlanta. *De mortuis nil—requiescant in pace.*

---

A DEATH from cocaine has just occurred in a dentist's chair in Goshen, Indiana. The patient was a man of fifty years of age, and was to have two teeth extracted. Ten minims of (nearly) an eleven per cent. solution were injected in the gums, and the patient died in a few minutes after the teeth were extracted. The toxic dose of cocaine in this case was therefore about one and one-twelfth of a grain.

---

UNFORTUNATELY for Georgia, the bill to regulate medical practice in that State has failed to become a law. This means that Georgia is likely to continue a particularly fertile field for cheap medical schools and cheap doctors.

The regular profession in that State may take heart, though, and profit by the advancement Kentucky has made on this line. The quacks here, like the Arab, are folding their tents and quietly stealing away. Patience, perseverance, and diligence.—*Medical Progress.*

PROFESSOR WILLIAM PEPPER has resigned his post as Provost of the Universty of Pennsylvania, on account of the press of other duties. His resignation was accompanied with his check for $50,000 for the improvement of the medical department of the University. He will retain the Chair of Practice, and devote more time to literary work. Dr. Pepper has already contributed much to medical literature, his *System of Medicine* and *American Text-Book* being the best known.

---

A LATE applicant before the Texas Examining Board was asked: "What is histology?" "Histology is the history of medicine." "What system of medicine do you practice?" "The Vanderbilt and St. Louis systems, and sometimes the *homonpath* system." "What is the homonpath system, as you call it?" "Sweatin' the patient."

In Texas and most other States such applicants are promptly declined and told to move on. They do move on to Georgia, Ohio, etc., where their ignorance is safely guarded by the absence of any law that may interfere with them.

---

The following gentlemen subscribed and paid for *The Journal* before leaving College in the spring and neglected to furnish their addresses. We would thank any one who would kindly let us know the desired addresses:

T. W. Bond, Southern Medical College.
D. J. Gunter, Southern Medical College.
H. H. Henderson, Atlanta Medical College.
W. W. Joiner, Atlanta Medical College.
G. S. Kelley, Atlanta Medical College.
E. L. Peek, Atlanta Medical College.
G. M. Yancey, Atlanta Medical College.

---

Dr. William Thomas Coggin, of Athens, Ga., performed the first American symphyseotomy in Freedman, Ala., March 12, 1892.

Dr. Coggin, in Heidelberg in 1890, heard of Morrisani's success and then decided to prefer symphyseotomy to craniotomy whenever a case should come under his care in which one or the other alternative should be presented. Dr. Coggin operated upon the wife of a miner, who had been fifteen and a half hours in labor and who had a contracted pelvis of the justo-minor type, computed to be one-third smaller than the average. After opening the symphysis he applied the forceps and delivered a male fetus weighing eleven and three-quarter pounds. It was noted that the pubic bones became separated two and three-quarter inches. There was no injury produced by the extraction of the child either to the sacro-iliac synchondrosis or to the soft parts. Under a proper restraining apparatus the pubes readily united; there was no lameness, and the mother and infant both did well.—*Buffalo Med. and Surg. Journal.*

---

The last few months have witnessed the death of some of the foremost medical men of Europe, among these Billroth in Germany, Charcot and Diday in France. The last is Brown-Séquard, who died in Paris, April 2d. He was born on the Island of Mauritius April 8, 1817. His father was an American sea-captain and his mother Mlle. Séquard. He adopted both surnames and became Charles Edward Brown-Séquard. He was educated in Paris where he graduated in medicine in 1840. From 1864 to 1869 and from 1873 to 1878 he resided in this country and lectured on Physiology at Harvard and at Bellevue Medical College. His life was devoted mainly to the study of physiology, and his experiments and contributions on this subject were numerous and of the highest scientific value. His last years were occupied largely in the study of animal extracts in the hope of finding some agent capable of prolonging life and of combating the usual weaknesses of age. His "elixir of life," so called by the secular press, was never realized, but his studies were the beginning of important investigations which promise to add largely to our therapeutic resources.

